# Correction: Synthesis of high surface area porous carbon from anaerobic digestate and it’s electrochemical study as an electrode material for ultracapacitors

**DOI:** 10.1039/d0ra90003f

**Published:** 2020-01-23

**Authors:** Vikash Chaturvedi, Saurabh Usgaonkar, Manjusha V. Shelke

**Affiliations:** Physical and Materials Chemistry Division, CSIR-National Chemical Laboratory Pune-411008 MH India mv.shelke@ncl.res.in; Academy of Scientific and Innovative Research (AcSIR) Ghaziabad-201002 UP India; Polymer Science and Engineering Division, CSIR-National Chemical Laboratory Pune-411008 MH India

## Abstract

Correction for ‘Synthesis of high surface area porous carbon from anaerobic digestate and it’s electrochemical study as an electrode material for ultracapacitors’ by Vikash Chaturvedi *et al.*, *RSC Adv.*, 2019, **9**, 36343–36350.

The authors regret that the name of one of the authors (Saurabh Usgaonkar) was shown incorrectly in the original article. The corrected author list is as shown above.

The Royal Society of Chemistry apologises for these errors and any consequent inconvenience to authors and readers.

## Supplementary Material

